# Predictors of time to recovery from postpartum hemorrhage in Debre Markos comprehensive specialized hospital, Northwest, Ethiopia, 2020/21

**DOI:** 10.1186/s12884-022-04834-5

**Published:** 2022-06-17

**Authors:** Bekalu Kassie, Beker Ahmed, Genet Degu

**Affiliations:** 1 Department of Midwifery, College of Health Sciences, Debre Markos University, Debre Markos, Ethiopia; 2 Department of Midwifery, College of Health Sciences, Arsi University, Assela, Ethiopia

**Keywords:** Debre Markos, Ethiopia, Postpartum hemorrhage, Time to recovery, Predictors

## Abstract

**Background:**

Postpartum hemorrhage is one of the leading causes of maternal deaths worldwide. Early recovery is a performance indicator and better health outcome of patients with postpartum hemorrhage. Therefore, this study aimed to assess time to recovery from postpartum hemorrhage and its predictors in Debre Markos Comprehensive Specialized Hospital, Ethiopia, 2020.

**Methods:**

A retrospective follow-up study was conducted among 302 women who were diagnosed with postpartum hemorrhage from January 1, 2016 to December 31, 2020 at Debre Markos Comprehensive Specialized Hospital. Consecutive sampling technique was employed. To show the statistical significant difference between each group of variables, log rank test was used. Kaplan Meier analysis to estimate time to recovery and cox proportional-hazard regression analysis to determine independent predictors were carried out cautiously. Adjusted hazard ratio used to determine the strength of association.

**Result:**

The median recovery time from postpartum hemorrhage was 13 h with range of (10 to 17 h). Blood transfusion (AHR: 1.8, 95% CI (1.39, 2.57)), NASG utilization (AHR: 6.5, 95% CI (4.58, 9.42)) fluid resuscitation (AHR 2.9, 95% CI (1.48, 5.54)), active management of third stage of labor (AHR: 1.7, 95% CI (1.18, 2.45)) and history of antenatal care follow-up (AHR: 2.6, 95% CI (1.91, 3.56)) were the predictors, which shorten the recovery time. Comorbidities like anemia at the time of admission (AHR: 0.62 95% CI (0.44, 0.89)), retroviral infection (AHR: 0.33, 95% CI (0.16, 0.67)) and Hepatitis B-Virus infection (AHR: 0.52, 95% CI (0.32, 0.82)) delay the recovery rate from postpartum hemorrhage.

**Conclusion:**

Mothers in North-West Ethiopia stays morbid from postpartum hemorrhage for more than half a day. Their recovery time was affected by Non-Pneumatic Anti-Shock Garment utilization, implementation of emergency management components like blood transfusion and fluid resuscitation, history of antenatal care follow up, and being comorbid with viral infections. Non-pneumatic anti-shock garment application to every mother with postpartum hemorrhage and implementation of proper emergency management approach are vital for rapid recovery from postpartum hemorrhage.

## Background

Postpartum hemorrhage (PPH) is a bleeding more than 500 ml after a vaginal delivery, 1000 ml after cesarean delivery and 1500 ml after hysterectomy blood loss. It is also defined as blood loss sufficient to cause hypovolemic or vital sign derangement, or a 10% drop in hematocrit [1]. It can be defined by considering both blood loss and clinical signs and symptoms of cardiovascular changes after childbirth [2]. Once a baby is delivered, the uterus normally contracts and pushes out the placenta and put pressure on the bleeding vessels in the region where the placenta was attached after it is delivered [3]. The causes of postpartum hemorrhage include poor uterine tone (uterine atony), retained placental tissue, abnormalities of placentation, genital tract trauma, and abnormalities of coagulation [4]. The leading cause of PPH is uterine atony (60–80%), followed by retained placenta and injury to genital tract [5]. Severe hemorrhage leads to serious maternal morbidity, which includes adult respiratory distress syndrome, renal failure, coagulopathy, shock, myocardial ischemia, hysterectomy, and long-term morbidity. About 20 million women worldwide are disabled, either acutely or chronically, due to excessive hemorrhage [6].

Long transports from home or primary health care facilities to the referral setup, lack of skilled providers, and lack of intravenous fluids and/or a safe blood supply often create long delays in instituting appropriate treatment. As a result, time to recovery from PPH delayed and the risk of maternal morbidity and mortality will increase [7–9]. Besides death and comorbidities, PPH is one of the most critical problems in the public health system that imposes substantial financial costs on the society [10, 11]. Postpartum hemorrhage occurs unpredictably, and no patient is immune from it. It is simply an equal opportunity killer [12].

Ethiopia has made significant progress on maternal health care facilities, including an improvement in institutional births, avail trained birth attendants at all births to reduce incidence of life-threatening comorbidities like PPH and the need for blood transfusions. Despite this, PPH remains the most common cause of maternal death. There are some studies on incidence and predictors of postpartum hemorrhage. However, the time to recovery from postpartum hemorrhage and its predictors remains unknown. Conducting research in this area is widely important to provide evidence based practice to enhance maternal health, reduce maternal morbidity and mortality, and also to improve the care in health care tier system. Therefore, this study was aimed to assess time to recovery from postpartum hemorrhage and its predictors in Debre Markos Comprehensive Specialized Hospital, North west, Ethiopia, 2021.

## Methods

### Study design, setting and period

A retrospective single cohort study design was conducted in Debre Markos comprehensive specialized Hospital (DMCSH), which is located in Derbre Markos Town, Northwest, Ethiopia. It was conducted among 302 mothers diagnosed with postpartum hemorrhage from January 1, 2016 until December 31, 2020. Derbre Markos Town is located about 299 km far from Addis Ababa, the capital city of Ethiopia and 265 km from Bihar Dar. DMCSH is providing service for more than 5 million people per year. In the maternity department, a total of 1 emergency surgeon 1MSc clinical midwife specialist, 41 general practitioners, 16 obstetrics and gynecology specialists and 44 midwives. The department has 60 beds for inpatient clients to serve high-risk mothers, gynecologic patients, and postnatal mothers. The annual delivery report showed that, 6017 mothers gave birth in the hospital.

#### Population

All women diagnosed with postpartum hemorrhage in Debre Markos comprehensive specialized hospital, during the period of Jan.1, 2016 to Dec. 31, 2020 G.C were taken as source and study populations. Clear record of diagnosis/PPH/ was used as an inclusion criteria. The diagnosis might be “anemia secondary to postpartum hemorrhage” or “hypovolemic shock/shock secondary to postpartum hemorrhage”. These diagnoses were included. Unclear anemia/shocks were excluded.

### Sample Size Determination

The sample size was calculated by using a sample size calculation for survival analysis in STATA 14. Sample size (n) = number of event / Probability of event.

$$\mathrm{Number of Event }=\frac{(\mathrm{Z a}/2+\mathrm{ ZB})2 }{\mathrm{pq }(\mathrm{logHR})2}$$

By taking the hazard ratio of Volume of IV fluids given in the first hour with NASG group (1.575) from a study conducted on time to recovery from shock for women with obstetrics hemorrhage in Egypt [2], the sample size was calculated. The assumptions for this were, probability of withdrawals (0.1), probability of developing an event (0.5), alpha (0.05), and power of (80%). Using STATA command (Stpowerlogrank 0.5, hratio (1.575) power (0.8) wdprob (0.1)), it becomes 302.

### Sampling technique

**Fig. 1:**
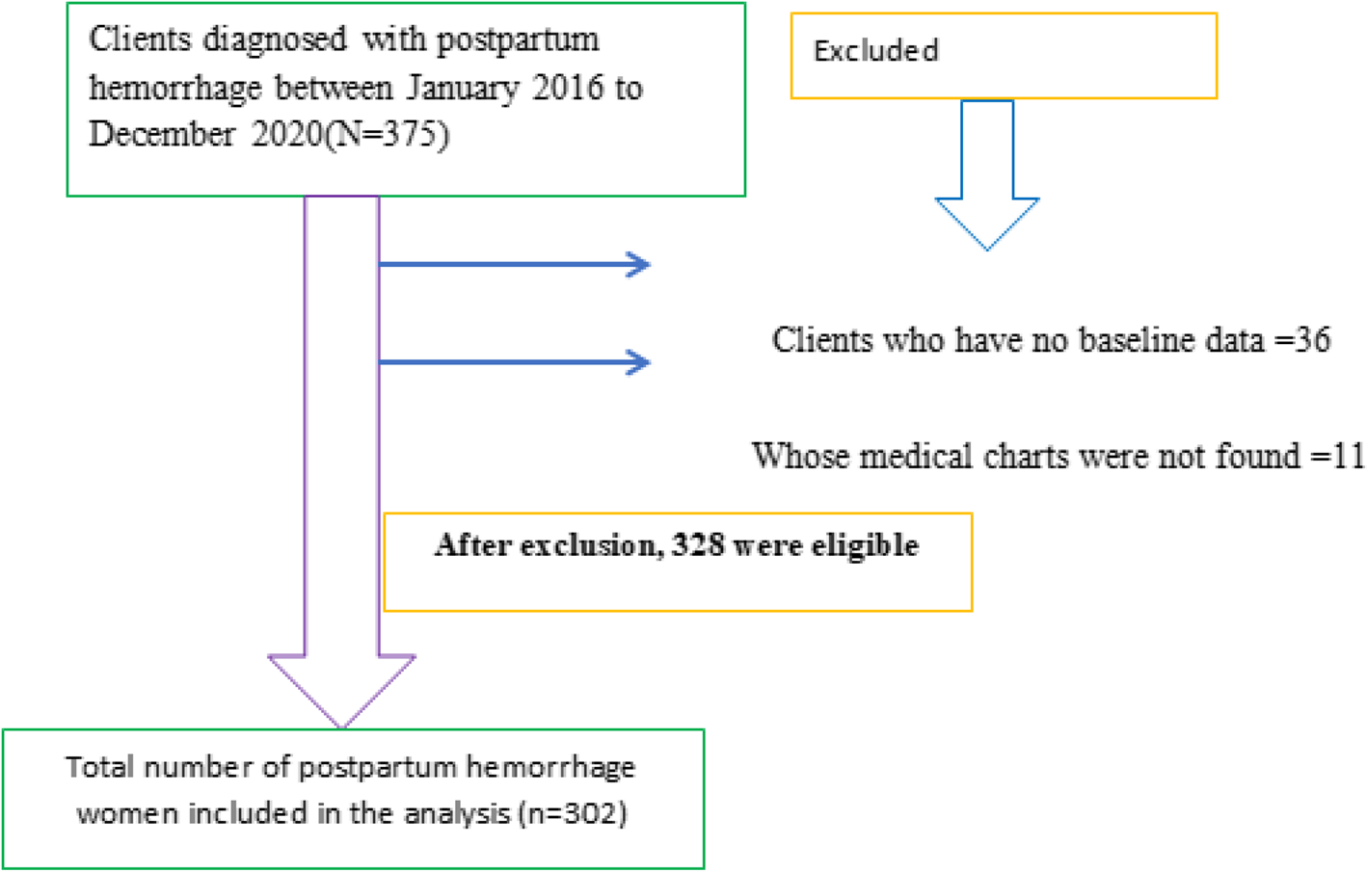
Diagrammatic presentation of the final sample size included in the study from January 1, 2016 to December 31, 2020, at Debre Markos comprehensive specialized hospital, Northwest, Ethiopia in 2020

By using registration book in maternity ward of DMCSH, maternal charts were retrieved and then data on individuals with postpartum hemorrhage was gathered starting from first date of diagnosis record up to the last record of the same diagnosis in the same period. All mothers with postpartum hemorrhage in the years 2016 to 2020 who fulfill inclusion criteria were consecutively included in the study. The total postpartum mothers in the hospital registered as anemia and or hypovolemic shock within the five years period were 375 and of these 328 fulfills the eligibility criteria. From these again 302 were selected consecutively because the total population was small to apply other probability sampling methods (Fig. 1).

### The Study variable

#### Dependent variable

Time to recovery from postpartum hemorrhage (in hours) (i.e., the event of interest is recovery).

### Independent variable

#### Socio-demographic

Age at diagnosis, marital status, residence, place of delivery.

#### Treatment-related variable

Fluid resuscitation, Blood transfusion, AMTSL (Active Management of Third Stage of Labor), NASG utilization, tranexamic acid utilization, MRP, Antibiotic used, types of surgery done.

#### Obstetrics related factors

Mode of delivery, complication of labor, Episiotomy, Birth weight, Gravidity, Parity, multiple pregnancy, antepartum iron intake, number of visits for ANC, status of anemia during the antepartum period, types PPH.

#### Comorbidities

HIV, TB, Syphilis, Hypertension, Hepatitis.

### Operational definition

#### Recovered

Women were considered as recovered when no more vital sign derangement, which means pulse rate < 100, and SBP ≥ 100 [13] and hematocrit greater than 21% after diagnosed for postpartum hemorrhage.

#### Start time

The time of diagnosis of PPH.

#### Follow-up period

Time of diagnosis to the first record of stable vital sign and/or hematocrit > 21%

#### Time to recovery

Was obtained by calculating the difference (in hours) from time of diagnosis until the last recorded deranged vital sign and/or last record of hematocrit <  = 21%

#### Event

The occurrence of stable vital sign and/or hematocrit > 21% after the diagnosis of postpartum hemorrhage.

#### Incomplete chart

Cards with no baseline data about diagnosis of PPH and when variables that affect the outcome variable in the study were missed such as, vital sign (pulse and blood pressure) and date of diagnosis.

#### Censored

Declared when the outcome of interest was not observed, like cases transferred to other facility.

#### Postpartum hemorrhage

Considered as PPH case if the health care provider diagnosed and recorded on the chart as PPH [14].

#### Co-morbidity

Is the presence of one or more diseases other than postpartum hemorrhage. The presence of any of these conditions during the follow-up was designated a “Yes‟, while the absence of these conditions was denoted as “No‟. Those comorbidities were accessed from the patient’s card.

#### Data collection tool

A structured data abstraction checklist was adapted from different literatures and standard treatment protocols for PPH.

#### Data collection procedure

The information was collected from the operation notes, admission and discharge delivery registration logbook, and patient cards. Data were extracted for one month by using a data extraction sheet that was prepared in the English language after reviewing different literatures and from the standard treatment for the management of PPH. Two midwives working in the maternity ward retrieved patients’ charts and extracted the required data under supervision.

#### Data quality assurances

To ensure the quality of data at the beginning, a data collection tool was pre-tested on 5% of the calculated sample size at Injibara General Hospital, which is found about 200 km from the actual study setting. Then, necessary changes were made based on the gaps identified. The abstraction format was checked to the hospital documentation system to make sure that the agreement of the data abstraction format with the need for the study. Any error found during the process was corrected and changes were made to the last version of the data abstraction format. Training on record review was given to the data collectors and supervisors for one day before the actual data collection. All collected data were reviewed for completeness and consistency during data cleaning and analysis.

### Data processing and analysis

Data were entered into Epi-Data version 4.2 after checking for completeness, and then the data were cleaned and transferred to STATA 14 statistical software for analysis. Schoenfeld residual analysis (global test) was used to show the cox proportional hazard model assumption validity. Predictors of time to recovery were identified using bivariable and multivariable cox proportional hazard models (CPHM). All independent variables that had a P value less than 0.25 in the bivariable analysis were considered as candidate variables for the multivariable analysis. The output of the multivariable CPHM was presented using adjusted hazard ratios with the respective 95% confidence intervals (CI) to measure the strength of association. Kaplan–Meier survival estimator was used to estimate median recovery time, while log-rank tests to compare time to recovery between groups. Before running the cox regression model, assumption of proportional- hazards and multi-collinearity were checked. The Cox Snell residual was used to assess model fitness and proportional hazard assumption.

### Ethical Considerations

An ethical approval letter was obtained from the Institutional Research Ethics Review Committee of college of health science, Debre Markos University. Following the approval, an official letter of cooperation was written to the concerned bodies by the college to facilitate the support and commitment of responsible bodies. Permission was obtained from the hospital medical director and department of gynecology and obstetrics/maternity. As the study was conducted through review of chart records, the individual patients were not subjected to any harm and personal identifiers during data collection and confidentiality was maintained through not recording the name of the participant on the checklist.

## Results

### Socio-demographic characteristics

Between January 1, 2016 to December 31, 2020, postpartum hemorrhage patient’s total charts (N = 375) were reviewed among which, 328 were eligible for this study. Of these, three hundred two postpartum hemorrhage women’s cards were included in the study. The median hemoglobin level was 6 g/dl while pulse rate and SBP were 120 bpm and 80 mmHg respectively. Nearly 277 (91.72%) patients were married; and 18.50% had a preexisting medical problem during diagnosis. More than two-third, 80.79%, of the participants were rural residents (Table 1).

**Table 1:** Socio-demographic and comorbidity characteristics of postpartum hemorrhage patients, Debre Markos Comprehensive specialized hospital, Northwest, Ethiopia, (*n* = 302)

Covariate	Category	Frequency (%)
Age in years	≤ 24	63(20.90)
	25–29	91(30.10)
	30–34	75(24.80)
	≥ 35	73(24.20)
Place of delivery	Home	51(16.90)
	Health institution	251(83.10)
HIV status	Negative	293(97.02)
	positive	9(2.98)
HBV	Yes	23(7.62)
	No	279(92.38)
Syphilis	Yes	14(4.64)
	No	288(95.36)

### Obstetrics characteristics

Among women with PPH, 72.20% had history of ANC follow-up. Regarding the antepartum obstetric event among women with PPH, about 6.62% had abruption placenta awhile 5.63% had previa. Majority of participants (98.70%) gave singleton birth, while the rest for twins. About 3.31% of mother encountered obstructed labor and 14.83% faced prolonged labor. There were also 23 (7.62%) preeclamptic mothers diagnosed with PPH. The predominant cause of PPH was uterine atony 183(65.6%) followed by retained placenta (41.4%) (Table 2).

**Table 2:** Obstetrics characteristics of postpartum hemorrhage patients at Debre Markos comprehensive specialized Hospital, Northwest, Ethiopia (*n* = 302)

Covariate	Category	Frequency (%)
Gravidity	One	41(13.58)
	Two-four	128(42.38)
	More than four	133(44.04)
Frequency of ANC (218)	≥ 4	121(55.50)
	< 4	97(44.50)
IFA supplementation	Yes	216(97.74)
	No	5(2.26)
Type of pregnancy	Singleton	298(98.68)
	Twin	4(1.32)
Mode of delivery	Spontaneous	265(87.75)
	Instrumental	19(6.29)
	Cesarean section	18(5.96)

### Treatment related variables

Regarding the management of the third stage of labor, 251(83.06) receives AMTSL namely uterotonic, cord traction, and uterine massage, while the rest did not receive. In most of the cases, 286 (97.7%) had adequate fluid resuscitation. NASG was applied for 128(42.3%) patients, and they were treated with antibiotics. Around sixteen (5.30) patient undergone major surgery with the indication of uncontrolled bleeding (Table 3).

**Table 3:** Treatment related characteristics of postpartum hemorrhage patients in Debre Markos Comprehensive specialized hospital, Northwest, Ethiopia (*n* = 302)

Covariate	Category	Frequency (%)
Transfused	Yes	231(76.49)
	No	71(23.51)
Unit of blood transfusion	One	110(47.62)
	Two	108(46.75)
	≥ 3	13(5.63)
NASG applied	Yes	56(18.54)
	No	246(81.46)
Antibiotics used	Yes	128 (42.38)
	No	174 (57.62)
Was Surgery done	Yes	16 (5.30)
	No	286(94.70)

From 302 patients with postpartum hemorrhage, 288(95.36%) were recovered from PPH in the health care setup. However, six (2%) died, and eight (2.64%) were transferred to another health care institution for further management.

#### Time to recovery from PPH

**Fig. 2:**
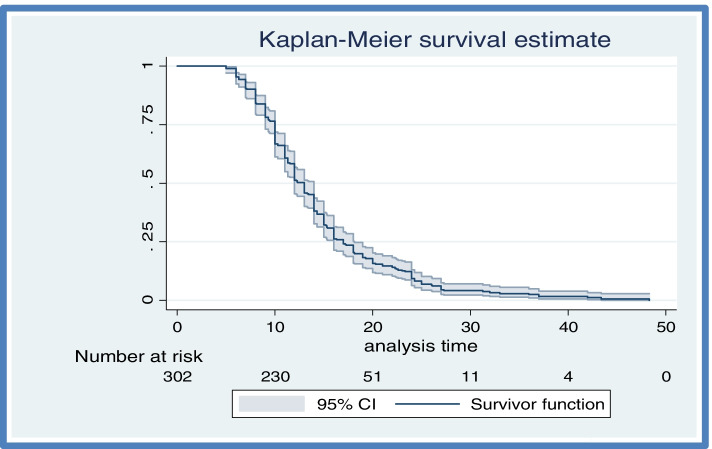
Overall median recovery time of the entire cohort patients’ diagnosed with PPH at Debre Markos Comprehensive Specialized Hospital, Northwest, Ethiopia, from January 1, 2016 to December 31, 2020

The overall median recovery time from postpartum hemorrhage in this study was 13 h (IQR = 10–17) (Fig. 2).

### Time to recovery among different groups of postpartum hemorrhage women

The log-rank test was executed to test the equality of survival curves for the presence of any significant differences in recovery time among various levels of the categorical variables considered in the study. Those categorical variables including comorbid by HIV, comorbid by HBV, history of ANC follow-up, fluid resuscitation, hemoglobin at diagnosis, blood transfusion, NASG utilization and active management third stage of labor. This significance difference was seen by the log-rank test. It is found that the median recovery time for those who delivered at home were 21 h, which is longer than the recovery time of those who delivered in health institution with 95% (16.02, 25.97). The median recovery time from PPH among participants living with HIV was longer (26.1 h, 95% CI: 8.27, 43.92) than those who were HIV negative (12.2 h, 95% CI: 11.53, 12.87). Among 302 participants, about 7.6% had HBV infection and their median recovery time from postpartum hemorrhage was 20 h (95% CI: 15.80, 24.19) (Table 4).

**Table 4:** Median recovery time of postpartum hemorrhage women by categorical variables from January 1, 2016 to December 31, 2020 (*n* = 302)

Covariate	Frequency		Survival time, hours, (95% CI)	Log rank test	*P*-value
Place of delivery	Home	51	21.00(16.02, 25.98)	34	0.000
	HI	251	12.00(11.29, 12.72)		
HIV	Negative	293	12.20(11.53, 12.87)	9.15	0.0025
	Positive	9	26.10(8.28, 43.92)		
HBV	Yes	23	20.00(15.81, 24.19)	14.33	0.0002
	No	279	12.00(11.31, 12.69)		
Hemoglobin at diagnosis	> 7 g/dl	42	11.50(10.72, 12.30)	12.84	0.0003
	≤ 7 g/dl	260	13.00(12.01, 13.93)		
ANC	Yes	218	11.30(10.66, 11.94)	69.33	0.001
	No	84	19.00(15.65, 20.36		
AMTSL	Yes	251	12.00(11.39, 12.61)	64.97	0.001
	No	51	20.30(17.92, 22.68)		
Blood transfusion	Yes	231	11.50(11.29, 12.71)	41.47	0.001
	No	71	19.00(13.35,18.65)		
Fluid resuscitation	Yes	286	12.00(11.33,12.67)	18.58	0.001
	No	16	23.00(15.27, 30.73)		
NASG utilization	Yes	56	8.00(7.48, 8.52)	17.72	0.001
	No	246	14.00(13.40, 14.59)		

The median recovery time for those who had ANC follow up was 11.3 h, (10.6, 11.93), which is shorter than those who had no ANC, 19 h (16.26, 21.74).

A log rank test conducted to indicate the time to recovery among mothers on whom NASG was applied and on who not applied was significantly different. The Kaplan–Meier graph along with log rank test shows that median recovery time for those who have utilized NASG had a shorter recovery time (8 h, 95% CI: 7.44, 8.56) than those who did not utilize (14 h, 95% CI: 13.40, 14.59).

### Testing proportional hazard assumption

**Fig. 3:**
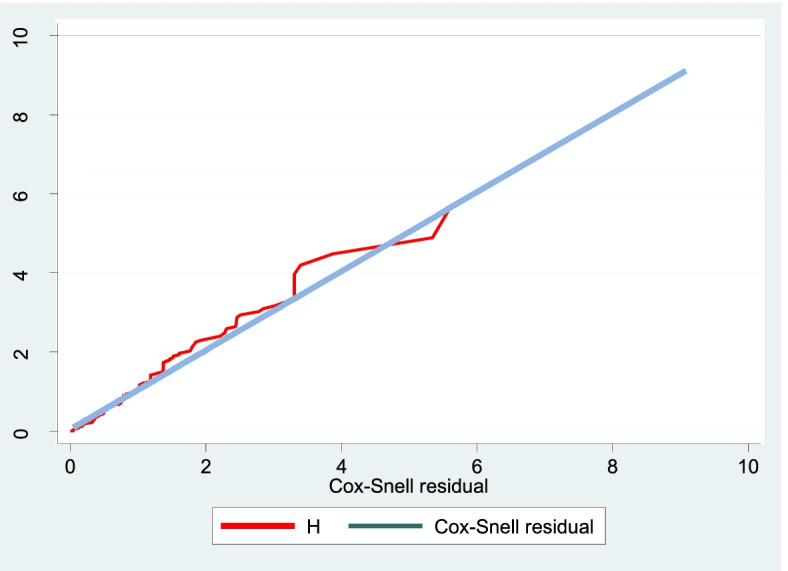
Cox-Snell residual Nelson -Aalen cumulative hazard graph on postpartum hemorrhage patients in Debre Markos comprehensive specialized hospital, Northwest, Ethiopia, and January 1, 2016 to December 31, 2020

A Cox regression model was used to examine the effects of socio-demographic, obstetrics and treatment characteristics of patients on time to recovery from PPH. A goodness-of-fit (GOF) test was conducted to assess the proportional hazard (PH) assumptions of the Cox model for a given predictor variable. This test was graphically supported (Fig. 3). The findings indicated that all variable included in the model satisfied PH assumptions (*p*-value > 0.05).

### Overall model fit

Since this graph shows a standard censored exponential distribution with HR of one and cox–Snell residuals were satisfied with the overall model fitness, the cox regression model fits the data. The global test of the proportional hazards assumption for this study was 0.26.

### Predictors of recovery from postpartum hemorrhage

#### Incidence of recovery

Patients were followed for a minimum of five and a maximum of 48.3 h, with 12 h of median follow-up time. Overall recovery rate was seven per 100 person-hours (95% CI: 0.06, 0.08) of observation.

In bivariable cox proportional hazard regression analysis, at 25% modest level of significance; place of delivery, AMTSL, comorbidity (HIV and HBV), blood transfusion, ANC follow-up, mode of delivery, uterine atony, episiotomy, cervical tear, fluid resuscitation, NASG application and hemoglobin at diagnosis were all statistically significantly associated with recovery time from PPH. Therefore, they were included in the multivariable analysis for further investigation.

In the multivariable cox regression analysis, those variables with *p*-value < 0.25 in the bivariate analysis and non-collinear independent variables were included. In the multivariable cox proportional hazards model, eight variables were associated with time to recovery from postpartum hemorrhage. The result of the multivariable analysis revealed that the rate of recovery among HIV positive women was reduced by 67% than negative women (AHR: 0.33, 95% CI: 0.16, 0.67). Similarly, the rate of recovery from PPH among mothers with HBV positive were 48% reduced than those with HBV negative (AHR: 0.52, 95% CI: 0.32, 0.82). Patients who had ANC follow were 2.7 times faster to recover as compared to those who have no ANC (AHR: 2.7, 95% CI: 1.96, 3.56).

Patient who were managed with AMTSL were 1.7 times more rapidly to recover as compared with those who managed without AMTSL (AHR: 1.7, 95% CI: 1.18, 2.45). Likewise, transfused patients were 1.8 times more likely to recover as compared to non-transfused ones (AHR: 1.8, 95% CI: 1.39, 2.57). Mothers on whom NASG was applied rate of recovery was 6.5 times quicker than their counterparts recovered (AHR: 6.3, 95% CI: 4.58, 9.42). Women who received fluid resuscitation had 2.9 times more likely to recover quickly as compared with women who did not receive (AHR: 2.9, 95% CI: 1.48, 5.54). Women with PPH who had hemoglobin below 7 g/dl at the time of diagnosis were 0.62 times less likely to recover as compared to those who had more than 7 g/dl (AHR:0.61, 95% 0.43, 0.89) (Table 5).

**Table 5:** Cox regression analysis of time to recovery from postpartum hemorrhage, Debre Markos Comprehensive specialized hospital, Northwest, Ethiopia, January 1^st^, 2016 to December 31.^st^, 2020(*n* = 302)

Characteristics	Category		Frequency	Recovered	Censored	CHR (95% CI)	AHR (95% CI)
HIV status		Negative	293	279	14	1	1
		Positive	9	9	-	0.38(0.19, 0.74)*	0.33(0.16, 0.67)**
HBV		No	279	267	12	1	1
		Yes	23	21	2	0.44(0.28,0.69)*	0.52(0.32, 0.82)**
Hemoglobin at diagnosis		≥ 7 g/dl	260	246	14	1	1
		< 7 g/dl	42	42	-	0.56(0.39, 0.78)	0.62(0.44, 0.89)
ANC follow up		No	84	79	5	1	1
		Yes	218	209	9	3(2.28, 3.99)	2.7(1.91, 3.56)**
AMTSL		No	51	47	4	1	1
		Yes	251	241	10	3.4(2.48, 4.79)*	1.7(1.18, 2.45)**
blood transfusion		No	71	67	4	1	1
		Yes	231	221	10	2.4(1.79, 3.15)	1.8(1.39, 2.57)**
Fluid resuscitation		No	16	12	4	1	1
		Yes	286	276	10	3.4(1.82, 6.19	2.9(1.48, 5.54)**
NASG utilization		No	246	233	13	1	1
		Yes	56	55	1	7.3(0.5.26, 10.49)*	6.5(4.58, 9.42)**

*CI* confidence interval, *AHR* Adjusted Hazard Ratio, *CHR* Crude Hazard Ratio, *NASG* Non-Pneumatic Anti-Shock Garment, *AMTSL* Active Management of Third Stage of Labor, *ANC* Antenatal Care, *HBV* Hepatitis B Virus,

^*^significantly associated with the outcome at bivariable analysis 95% level of significant (< 0.25). ** Significant (*p* < 0.05)

## Discussion

This study aimed to assess time to recovery and predictors among PPH patients. At the end of follow-up, about 288 patients were recovered and 14 were censored. The overall recovery rate in this study was seven per 100 person-hours with (95% CI: 0.06–0.08) of observation. Variables such as, ANC follow up, fluid resuscitation, blood transfusion, NASG utilization, AMTSL, hemoglobin at diagnosis, HIV, and HBV were predictors of time to recovery. HIV positive patients had a 67% delayed chance of recovery when compared with those without HIV. Recovery was delayed among women with HBV positive. Even though the mechanism through which those comorbidities delay the recovery rate is not yet known, some scholars suggested that the increased thrombocytopenia that is observed in 10 to 30% of HIV positive persons might be the one responsible for hemorrhage. Another possible reason for delay from recovery were immune suppressive effects, increases energy consumption, induces malabsorption, predisposes for loss of immunity, and again it results in inability to respond to standard treatments.

Women who receive fluid had faster recovery as compared with women who did not receive fluid. This finding was supported by WHO recommendation as fluid establishes hemodynamic stability, and improves oxygen-carrying capacity and preserves tissue oxygenation [15]. Blood transfusion was another significant predictor of time to recovery from PPH. The rate of recovery from PPH was faster among women transfused with blood as compared with no blood transfusion. One unit of packed RBCs should raise the hemoglobin of an average adult by 1 g/dL and the hematocrit by 3%. Blood products are also used to support hemostasis and correct coagulopathy. Blood transfusion maintains vital organ perfusion till about 30% of total blood loss [16].

Another covariate that had a significant effect on a median time to recovery was implementation of AMTSL. The rate of recovery from PPH among women who utilized AMTSL were 1.7 times more as compared without AMTSL. WHO recommends AMTSL which comprises administration of intramuscular uterotonic drugs to reduce the amount of blood loss in atonic PPH [17], which means recovery rate can be enhanced by uterotonic drug administration.

The rates of recovery from PPH among women who had ANC follow-up was faster than who had not. This might be because during ANC follow-up, iron is routinely supplemented that increases the level of hemoglobin to prevent risk of complication in postpartum bleeding. The other reason may be, during ANC follow-up, women can have the chance to get early screening and detection of risk factors for PPH [18].

NASG application is an important, strong predictor of recovery from PPH. This study showed that patients who utilized NASG were 6.5 times more likely to recover from postpartum hemorrhage as compared to those who did not. This finding is supported by studies done in Colombia [19] and Egypt [20]. This device applies circumferential counter pressure to the lower part of the body. Thus, it decreases the total vascular space in the lower portion of the body, while simultaneously increasing the volume of the blood in the central circulation. Therefore, it facilitates early recovery [21]. Women who have hemoglobin below or equals to 7 g/dl at the time of diagnosis were 0.61 times less likely to recover from postpartum hemorrhage as compared to those who have hemoglobin above 7 g/dl. The major function of hemoglobin is the transportation of oxygen from lungs to all the tissues of the body.

## Conclusion

Overall, recovery rate from PPH in this study was seven per 100 person-hours (95% CI: 0.06, 0.08) of observation. The median recovery from postpartum hemorrhage was 13 h (IQR 10, 17). According to this study, women who utilized NASG had a faster recovery rate as compared with those who did not. On the contrary, women with comorbidities like HIV, HBV, and hemoglobin below 7 g/dl had delayed recovery time. This study provides further evidence on the role of NASG utilization, fluid resuscitation, blood transfusion, AMTSL and ANC follow-up in predicting the time to recovery from PPH.

### Recommendations

Special emphasis should be given to patients with low hemoglobin at the time of diagnosis and comorbidities. The health care provider should implement routine AMTSL management and initiate fluid and blood transfusion as early as possible to prevent complications of PPH due to delay from recovery. Utilization of non-pneumonic anti-shock garment/NASG/ should be implemented for every one with excessive blood loss to facilitate early recovery.

### Limitation of the study

Since this study is a retrospective type, it is poor in measuring the actual time that when the woman recovered from complications of PPH. Additionally, missing variables due to poor recording system of health care institutions, not comparing the finding with other similar studies due to lacking such articles are limitations of this study.

## Data Availability

Even though the datasets generated and/or analyzed during the current study are available from the corresponding author, it is not publicly available due to the ethical principles of the study. We gave promise to the study participants’ representative not to disclose the dataset to others other than the authors.
